# Research focus and theme trend on fulminant myocarditis: A bibliometric analysis

**DOI:** 10.3389/fcvm.2022.935073

**Published:** 2022-09-14

**Authors:** Weimei Yang, Xifei He, Zhaozhao Wang, Lijuan Lu, Ge Zhou, Jie Cheng, Xinying Hao

**Affiliations:** ^1^Department of Cardiovascular Diseases, Tongji Hospital Affiliated to Tongji Medical College, Huazhong University of Science and Technology, Wuhan, China; ^2^School of Humanities and Social Sciences, University of Science and Technology of China, Hefei, China

**Keywords:** fulminant myocarditis (FM), bibliometric analysis, research focus, theme trends, citation network

## Abstract

**Aims:**

This study intends to explore the research focus and trends of fulminant myocarditis (FM) to have a better understanding of the topic.

**Materials and methods:**

The data were downloaded from the Web of Science (WoS) database using the topic (TS) advanced search strategy. Many instruments were used to extract, analyze, and visualize the data, such as Microsoft Excel, HistCite Pro, GunnMap, BibExcel, and VOSviewer.

**Results:**

From 1985 to 2022, 726 documents were indexed in the WoS. The United States and Columbia University were the most productive country and institutions. Keywords co-occurrence was carried out and four research themes were identified. In addition, the top three prolific authors, the first three highly cited authors, and the core authors of the author co-citation network were identified. The topics that they kept an eye on were analyzed, and the research areas of key authors were similar to the results of keyword co-occurrence. The hot topics of FM were related to the mechanical circulatory support, etiology, diagnosis, and the disease or therapy associated with FM.

**Conclusion:**

This study carried out a systematic analysis of the documents related to FM from 1985 to 2022, which can provide a guideline for researchers to understand the theme trend to promote future research to be carried out.

## Introduction

Myocarditis can be divided into two categories, fulminant myocarditis (FM) and non-fulminant myocarditis (NFM), which is based on the medical history, clinical manifestation, physical examination, examination result, and test result (1). FM is the most serious and special type of myocarditis, which is characterized by its acute and explosive nature (1, 2). FM is a sudden-onset and life-threatening disease and has a high fatality rate. The study indicated that the mortality rate of FM was more than 50%, though FM only occupied approximately 10–38% of all cases of acute myocarditis (2–4).

Fulminant myocarditis can be caused by viruses, bacteria, and non-infectious origins, and the virus is the main etiology (2). Several specific conditions may result in FM, such as lymphocytic myocarditis, giant cell myocarditis, eosinophilic myocarditis, and immune checkpoint inhibitors (ICI)-associated myocarditis (3, 5, 6). Coronavirus has been also reported to be associated with FM. For example, studies showed that OC43 subtype coronavirus and coronavirus disease (COVID-19) are related to FM (7, 8). Besides, studies indicated that the influenza A (HINI) pandemic was related to a high prevalence of FM (9, 10).

Fulminant myocarditis is difficult to diagnose, because the manifestations are often atypical, such as chest pain, arrhythmia, and dyspnea (11). In addition, most patients with FM are often younger and healthier and are easy to be ignored (3). FM can lead to cardiovascular damage and ventricular dysfunction (1, 12), presenting with cardiogenic shock, progressive hemodynamic, and fatal arrhythmia (13, 14). The above manifestations may occur about 2 weeks after virus infection, which may be life-threatening for patients (3, 15). The patients can be rescued if they receive therapy in time. For example, mechanical circulatory support is an effective therapy for FM (2, 16). Early recognition, diagnosis, and treatment are very important, which is the basis of favorable outcomes (17). It is important to note that although the early mortality of FM is high, the long-term prognosis of FM is still controversial. Some studies reported that the long-term prognosis is better than patients with acute myocarditis once patients pass the acute crisis. For example, an 11-year study found that patients with FM had a 93% survival rate if they did not receive heart transplantation, while the survival rate of the patients with acute myocarditis was only 45% (12). But some studies show that the outcome of the FM is worse than the NFM (18). For example, a 7-year study found that patients diagnosed with FM with left ventricular systolic dysfunction have higher rates of cardiac death and heart transplantation when compared with patients with NFM. In the future, the researchers need to carry out more parallel studies to explore the outcome difference between FM and NFM, maybe the subtype of FM.

There were reviews of FM, and they were mainly concentrated on the etiology, diagnosis, therapy, or outcome of FM (19, 20). In addition, they often paid attention to a specific topic of FM and did not present an overall result of FM research for readers. As researchers pay more attention to FM, the number of documents published has been increasing, and the research topics have been enriched. Therefore, to understand the research trend of FM deeply, a comprehensive literature analysis needs to be carried out from a bibliometric perspective. Bibliometric analysis is an effective way to explore and analyze large volumes of scientific data (21), and it has been used in various areas (22–24). In this study, the bibliometric method will be used to gain a better understanding of the research on FM based on the Web of Science (WoS) core collection database, which can provide a reference for researchers in the future.

## Materials and methods

**FIGURE 1:**
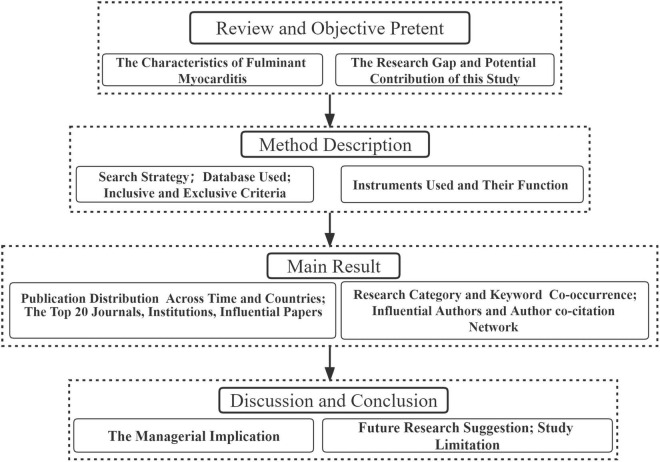
Flowchart of the methodology.

To analyze and visualize the data collected, Microsoft Excel software, HistCite Pro, GunnMap 2, BibExcel, and the Java program VOSviewer software were used. The workflow of the methodology is shown in Figure 1.

### Search strategy

Bibliometric data can be obtained through diverse search engines, such as the WoS database, Scopus database, and PubMed database. The WoS database was used as the scientific research output in this bibliometric study, as it is one of the largest, most reliable, complete, and comprehensive bibliographic databases covering multidisciplinary areas (25). The 2021 JCR covers 26,696 journals across 254 categories, spanning 118 countries.

In this study, information about scientific output was extracted from 6 databases in the Web of Science Core Collection [Science Citation Index EXPANDED (SCI-EXPANDED), Social Sciences Citation Index (SSCI), Conference Proceedings Citation Index-Science (CPCI-S), Conference Proceedings Citation Index-Social Science & Humanities (CPCI-SSH), Current Chemical Reactions (CCR-EXPANDED), and Index Chemicus (IC)]. The key topic used for retrieval in this study is “TS = ((‘Fulminant’) AND (‘Myocarditis’)).”

#### Inclusive and exclusive criteria

English is a universal language and is easily understood and retained. Therefore, this study has included documents published in English only, and the rest have been excluded from the survey. The majority of documents are either articles or conference proceedings, and it would not make much difference to leave others, so document type has no exclusion. Other parameters, such as open access, author name, subject area, affiliation, funding sponsor, and country, should also not be used to exclude any other document, because they do not affect the selection criteria (26).

To ensure the systematic and accurate retrieval of the article, the search strategy and inclusive and exclusive criteria were determined by the research team member. In addition, this study has carried out a detailed survey of publications related to FM according to inclusive and exclusive criteria by two researchers. If there were any different opinions, the third researcher would participate in the discussion. Finally, 726 documents about FM were collected from its inception to 5 January 2022.

### Data analysis and visualization

First, the text format documents containing all the bibliometric information were downloaded from the WoS database. Then, the data analysis and visualization were carried out using various instruments, including the Microsoft Excel software, HistCite Pro, GunnMap 2,^1^ BibExcel, and the Java program VOSviewer software. HistCite is a software developed to allow researchers to visualize the results of literature searches on the WoS. It helps the users analyze and organize the results of a search to obtain various views of the topic’s structure, history, and relationships (27, 28). BibExcel is a great tool for helping with bibliometric analysis and citation studies in particular (29–31). BibExcel can realize the data extraction, case conversion, synonym disambiguation, formation of the co-word matrix, and visualization of network files. VOSviewer is a software tool for constructing and visualizing bibliometric networks (24, 32). In this study, the Microsoft Excel software and HistCite Pro were used to obtain information concerning publication year, countries, annual output, journals, institutions, authors, language, and most cited documents. GunnMap 2 was selected to depict the world map, presenting publication distribution. In addition, BibExcel and VOSviewer were used to visualize and analyze the co-occurrence network of keywords extracted from the article, the collaboration networks between countries, and co-citation network relationships among authors.

### Keyword co-occurrence network

Keyword co-occurrence network (KCN) refers to the statistics and displaying of keywords in the literature, focusing on uncovering the knowledge components and structure of a certain research field by analyzing the link between keywords (26, 33). All keywords were included in this study, including author keywords and keywords plus, and KCN was analyzed by BibExcel and VOSviewer.

#### Author co-citation analysis

Author co-citation analysis (ACA) defines two authors as co-cited when at least one document from each author’s oeuvre occurs in the same reference list and their co-citation count as the number of different publications that co-cite them in this sense. Ever since it was introduced by White and Griffith in 1981, ACA has been a primary research tool for the study of the intellectual structure of research fields and the social structure of the underlying communities of researchers (34). ACA can be carried out by many software, such as Citespace, NetDraw, Gephi, BibExcel, and VOSviewer. In this study, ACA was achieved by analyzing the references through the BibExcel and VOSviewer software.

## Result

The most prolific countries, journals, institutions, authors, and main research category concerning FM and the influential articles in this area were displayed in this part.

### Descriptive analysis

After retrieval, 726 documents about FM, indexed in the WoS from 1985 to 2021, were included in this study. The literature included 685 articles (94.35%), 27 proceedings papers (3.72%), 12 early access (1.65%), and 1 book chapter (0.14%).

#### Publication distribution across time and countries

**FIGURE 2:**
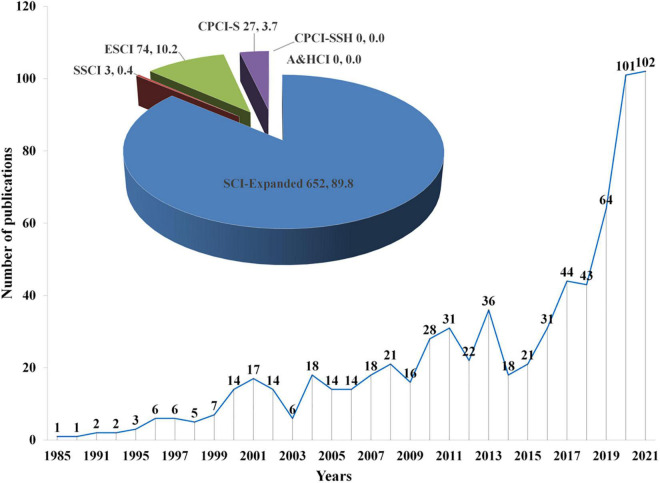
Growth trend of the Web of Science documents “fulminant myocarditis” research over the last 30 years.

Figure 2 shows the annual publications from 1985 to 2021 (by 5 January 2022). It was clear that there were limited publications before 2000. The documents began to increase steadily during the period from 2014 to 2021, and documents have grown rapidly since 2019, reaching a peak (102) in 2021. This indicates that the research community has had an increasing interest in FM in recent years. There were 652 (89.8%) documents indexed in the SCI-Expanded, 3 (0.4%) documents indexed in the SSCI, 74 (10.2%) documents indexed in the ESCI, and 27 papers indexed in the CPCI-S (3.7%). No article was indexed in A&HCI and CPCI-SSH.

**FIGURE 3:**
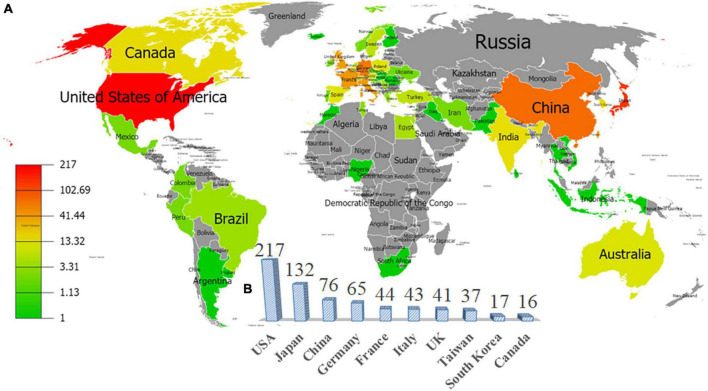
**(A)** Global geographic distribution of the total number of documents by country and **(B)** the top 10 prolific countries based on the number of publications related to “fulminant myocarditis” research.

**FIGURE 4:**
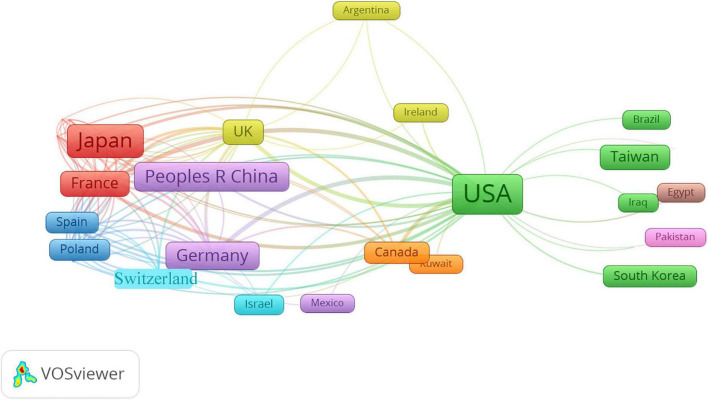
Network visualization map of country co-authorships. Thicker lines indicate stronger collaborations. Countries represented with larger circle size or font size had relatively more publications.

The data indicated that 71 countries/regions participated in research on FM. Figure 3 is a world map with productive countries based on the total number of documents on this theme. There were 49 countries that published only 1–5 articles and 22 countries that published at least 7 articles. The color of each pattern represents the total number of articles published. The more reddish the color of the pattern, the higher the number of total articles (TA) was. The United States, with 217 publications, was the most prolific country in producing articles related to FM, followed by Japan (132 publications), China (76 publications), Germany (65 publications), and France (44 publications). The 22 most productive countries generated 809 articles (111.4%), which indicated that there existed cooperation among nations. To find out the international collaboration networks among countries, country co-authorship network visualization was introduced. Figure 4 shows the collaboration network of countries that had at least one article. The nodes of the United States, Italy, Germany, the United Kingdom, France, and Switzerland were the biggest, which means they had the closest collaboration with other countries. The United States was the most networked country, collaborating with 111 countries, followed by Italy (*n* = 62), Germany (*n* = 59), the United Kingdom (*n* = 59), France (*n* = 48), and Switzerland (*n* = 32). This shows that many countries attach great importance to FM and have formed close international cooperation networks, especially in developed countries, which may be related to their relatively advanced medical systems.

#### Publication analysis based on journals

The 322 source journals publishing articles concerning FM were identified in this study. To evaluate the journals, five major measurement indexes were included in this study, such as the journal impact factor (JIF), TA, and total global citation score (TGCS). The top 20 productive journals are shown in Table 1, which published 231 articles and accounted for 31.8% of the TA on this topic. Circulation Journal (22, 3.0%) was the most prolific journal in this field, followed by the Journal of Heart and Lung Transplantation (19, 2.6%), Annals of Thoracic Surgery (17, 2.3%), and ESC Heart Failure (17, 2.3%). Journals of the American College of Cardiology and Circulation also played an important role because of the huge number of GCSA (153.6) and JIF (29.7), respectively. Besides, there were 12 journals with the JIF not less than 2.5, accounting for 60% of the top 20 prolific journals. In addition, 10 of the top 20 productive journals were from the United States, the others were from Japan, England, Australia, Switzerland, Ireland, and Germany.

**TABLE 1:** Quantitative measurements of journals publishing documents concerning “fulminant myocarditis” research.

Rank	Source title	JIF2020 (R)	TA (%)	TGCS	GCSA	JC
1	Circulation Journal	3.0	22 (3.0%)	572	26.0	Japan
2	Journal of Heart and Lung Transplantation	10.2	19 (2.6%)	499	26.3	United States
3	Annals of Thoracic Surgery	4.3	17 (2.3%)	568	33.4	United States
4	Esc Heart Failure	4.4	17 (2.3%)	47	2.8	England
5	Pediatric Cardiology	1.7	14 (1.9%)	260	18.6	United States
6	Internal Medicine	1.3	13 (1.8%)	76	5.8	Japan
7	Journal of Artificial Organs	1.7	13 (1.8%)	74	5.7	Japan
8	ASAIO Journal	2.9	11 (1.5%)	187	17.0	United States
9	Japanese Circulation Journal-English Edition	1.4	11 (1.5%)	126	11.5	Australia
10	American Journal of Emergency Medicine	2.5	10 (1.4%)	37	3.7	United States
11	Artificial Organs	3.1	10 (1.4%)	153	15.3	United States
12	European Heart Journal-Case Reports	–	10 (1.4%)	26	2.6	England
13	Herz	1.4	10 (1.4%)	161	16.1	German
14	Circulation	29.7	8 (1.1%)	577	72.1	United States
15	International Heart Journal	1.9	8 (1.1%)	54	6.8	Japan
16	International Journal of Cardiology	4.2	8 (1.1%)	95	11.9	Ireland
17	Medicine	1.9	8 (1.1%)	10	1.3	United States
18	Pediatric Critical Care Medicine	3.6	8 (1.1%)	42	5.3	United States
19	Frontiers in Cardiovascular Medicine	6.1	7 (1.0%)	1	0.1	Switzerland
20	Journal of the American College of Cardiology	24.1	7 (1.0%)	1,075	153.6	United States

JIF, journal impact factor; TA, total articles; TGCS, total global citation score; GCSA, global citation score per article; JC, journal country.

#### Publication analysis based on institutions

The top 20 productive institutions, based on the number of documents concerning FM, are illustrated in Table 2. In terms of the number of articles published, Columbia University (*n* = 15, 2.1%) ranked first, followed by Huazhong University Science and Technology (13 articles, 1.8%), and National Cerebral and Cardiovascular Center (12 articles, 1.7%). By the way, although some institutes with fewer articles, the TGCS was high. For example, from the perspective of TGCS, Johns Hopkins University was in first place among the top 20 productive institutions, even though the number of articles only ranked eighteenth. Besides, the top 20 most active institutions were from 5 countries, namely, the United States, Japan, China, Italy, and Germany. Notably, 50% of the top 20 institutions were from the United States.

**TABLE 2:** Quantitative measurements of institutions publishing articles concerning “fulminant myocarditis” research.

Rank	Institution	Country	TA	TA (%)	TGCS	GCSA
1	Columbia University	United States	15	2.1	296	19.7
2	Huazhong University Science and Technology	China	13	1.8	69	5.3
3	National Cerebral and Cardiovascular Center	Japan	12	1.7	136	11.3
4	Ospedale Niguarda Ca’ Granda	Italy	12	1.7	516	43.0
5	Harvard Medical School	United States	11	1.5	1574	143.1
6	Massachusetts General Hospital	United States	11	1.5	2196	199.6
7	Mayo Clinic	United States	11	1.5	362	32.9
8	Fujita Health University	Japan	10	1.4	242	24.2
9	University Texas MD Anderson Cancer Center	United States	10	1.4	1518	151.8
10	Vanderbilt University	United States	10	1.4	1851	185.1
11	Chang Gung University	China Taiwan	9	1.2	145	16.1
12	Cleveland Clinic	United States	9	1.2	228	25.3
13	Harvard University	United States	9	1.2	374	41.6
14	Kitasato University	Japan	9	1.2	344	38.2
15	Nagoya University	Japan	9	1.2	110	12.2
16	University of Pennsylvania	United States	9	1.2	460	51.1
17	Charite – University Medicine Berlin	Germany	8	1.1	161	20.1
18	Johns Hopkins University	United States	8	1.1	2279	284.9
19	Mie University	Japan	8	1.1	155	19.4
20	Kyoto University	Japan	7	1.0	298	42.6

TA, total articles; TGCS, total global citation score; GCSA, global citation score per article.

### Research-focused analysis by the co-occurrence of keywords and research category

**FIGURE 5:**
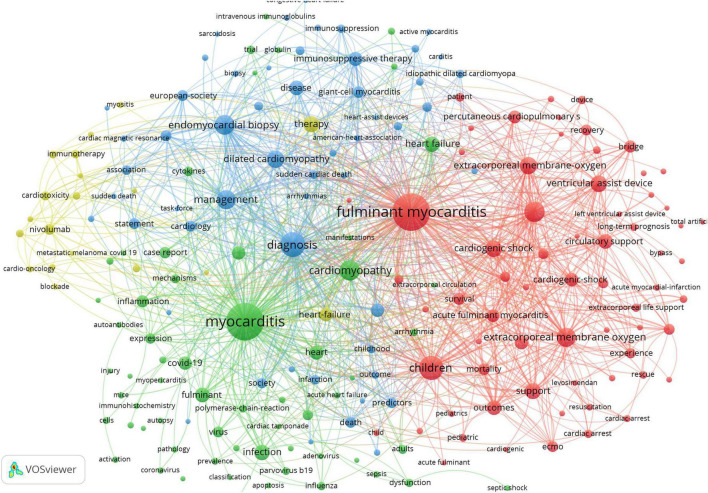
Co-occurrence network of the terms with occurrences of 5 or more extracted. The larger circle size or font size indicates a higher occurrence.

**FIGURE 6:**
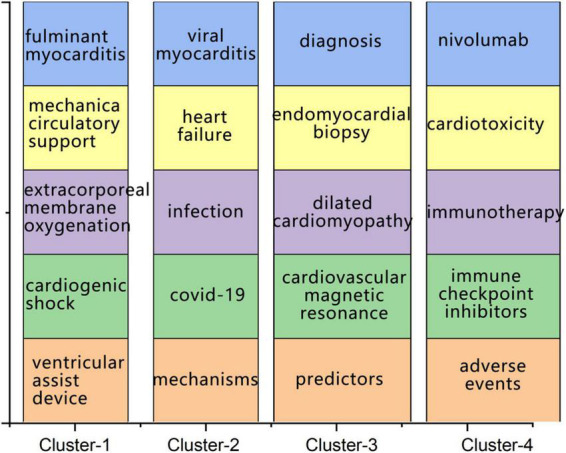
The top five representative keywords of each cluster.

Keywords can reflect the main content of a research area. Therefore, to better understand research priorities and trends, keyword co-occurrence analysis is often performed (25). In this study, keywords with occurrences of five or more were extracted and analyzed. Finally, 201 terms met the inclusion criteria, and the occurrence result is shown in Figure 5. The result showed that there were four clusters, including red cluster (cluster 1), green cluster (cluster 2), blue cluster (cluster 3), and yellow cluster (cluster 4), and the top five representative keywords of each cluster were presented in Figure 6. In addition, the research primarily concentrated on the etiology, diagnosis, and therapy of FM.

**FIGURE 7:**
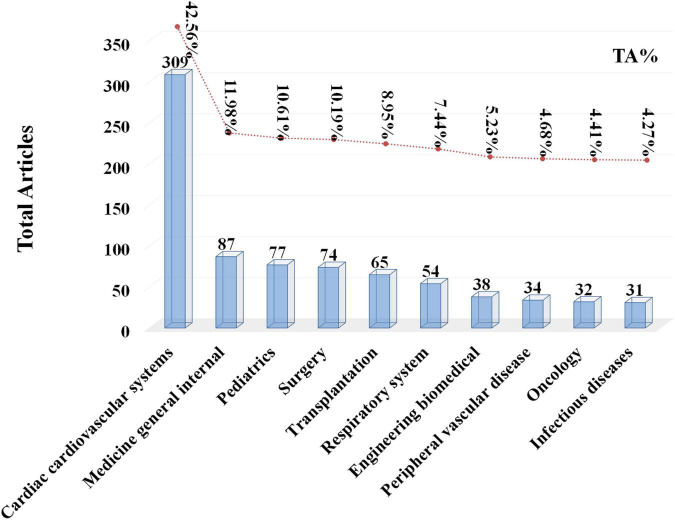
The top 10 research categories ranked by count.

To better understand the research area, the top 10 research categories (seen in Figure 7) were analyzed. The cardiac cardiovascular system (309, 42.56%) ranked first, followed by medicine general internal (87, 11.98%), pediatric (77, 10.61%), and surgery (74, 10.19%).

### Analysis of top 20 cited articles

The top 20 cited articles, based on total citation (TC), were identified and are shown in Table 3. Among the top three cited articles, two of them were focused on ICI-associated FM (6, 35). The third most cited article was about the outcome difference between FM and NFM, published in 2000 (36). Of the 20 articles, four concentrated on ICI-associated FM (6, 35, 37, 38), published from 2016 to 2018. Of the top 20 cited articles, six were about mechanical support for patients diagnosed with FM (39–44), published from 2001 to 2013. In addition, the 19th article was also about mechanical support, which focused on the application of venoarterial extracorporeal membrane oxygenation (ECMO) in adults, published in 2019 (45). Of the top 20 cited articles, two focused on the outcome or echocardiographic characteristics between FM and NFM (36, 46), both published in 2000. Notably, two articles focused on the COVID-19-associated FM (47, 48), published in 2020. Of the top 20 cited articles, three were about clinicopathological description, recognition, and management of myocarditis (3, 49, 50), and they were ranked 7th, 15th, and 18th, respectively. The 7th article was published in 1991 (49), the 15th article was published in 2020 (3), and the 18th article was published in 2019 (39). The other two articles explored the relationship between enterovirus infection and myocarditis (36) and the management of myocarditis induced by clozapine (37), respectively.

**TABLE 3:** Top 20 cited articles on “fulminant myocarditis” research from the inception of the searched databases to 2022.

Rank	Article	Author(s)	Journal	TC	TC/Y
1	Management of immune-related adverse events in patients treated with immune checkpoint inhibitor therapy: American society of clinical oncology clinical practice guideline (35)	Brahmer et al.	Journal of Clinical Oncology	1,327	265.4
2	Fulminant myocarditis with combination immune checkpoint blockade (6)	Johnson et al.	New England Journal of Medicine	926	132.29
3	Long-term outcome of fulminant myocarditis as compared with acute (non-fulminant) myocarditis (36)	McCarthy et al.	New England Journal of Medicine	502	21.83
4	Outcomes and long-term quality-of-life of patients supported by extracorporeal membrane oxygenation for refractory cardiogenic shock (40)	Combes et al.	Critical Care Medicine	409	27.27
5	Myocarditis in patients treated with immune checkpoint inhibitors (38)	Mahmood et al.	Journal of The American College of Cardiology	363	72.6
6	Cardiovascular toxicities associated with immune checkpoint inhibitors: an observational, retrospective, pharmacovigilance study (37)	Salem et al.	Lancet Oncology	294	58.8
7	Clinicopathological description of myocarditis (49)	Lieberman et al.	Journal of The American College of Cardiology	232	7.25
8	First case of COVID-19 complicated with fulminant myocarditis: a case report and insights (47)	Zeng et al.	Infection	216	72
9	Echocardiographic findings in fulminant and acute myocarditis (46)	Felker et al.	Journal of The American College of Cardiology	207	9
10	Emergency circulatory support in refractory cardiogenic shock patients in remote institutions: a pilot study (the cardiac-RESCUE program) (39)	Beurtheret et al.	European Heart Journal	173	17.3
11	Mechanical circulatory support for the treatment of children with acute fulminant myocarditis (41)	Duncan et al.	Journal of Thoracic and Cardiovascular Surgery	158	7.18
12	Outcomes, long-term quality of life, and psychologic assessment of fulminant myocarditis patients rescued by mechanical circulatory support (42)	Mirabel et al.	Critical Care Medicine	133	11.08
13	Is there an association between COVID-19 mortality and the renin-angiotensin system? A call for epidemiologic investigations (48)	Hanff. et al.	Clinical Infectious Diseases	129	43
14	Favourable clinical outcome in patients with cardiogenic shock due to fulminant myocarditis supported by percutaneous extracorporeal membrane oxygenation (43)	Asaumi et al.	European Heart Journal	123	6.83
15	Recognition and initial management of fulminant myocarditis: A scientific statement from the American Heart Association (3)	Kociol et al.	Circulation	117	39
16	An outbreak of enterovirus 71 infection in Taiwan 1998: A comprehensive pathological, virological, and molecular study on a case of fulminant encephalitis (51)	Yan et al.	Journal of Clinical Virology	115	5
17	A new monitoring protocol for clozapine-induced myocarditis based on an analysis of 75 cases and 94 controls (52)	Ronaldson et al.	Australian and New Zealand Journal of Psychiatry	110	9.17
18	Inflammatory cardiomyopathic syndromes (50)	Trachtenberg et al.	Circulation Research	105	17.5
19	Venoarterial ECMO for adults: JACC scientific expert panel (45)	Guglin et al.	Journal of The American College of Cardiology	104	26
20	Experience and result of extracorporeal membrane oxygenation in treating fulminant myocarditis with shock: What mechanical support should be considered first? (44)	Chen et al.	Journal of Heart And Lung Transplantation	99	5.5

TC, total citation; TC/Y, average citations per year, which is the TC divided by the year.

Among the top 20 articles, 11 were published earlier and were all before 2015, which may be contributed to the high citation. The other nine articles were published latest, but they were also paid high attention to. In addition, this can be illustrated for different reasons. First, four articles were about ICI-associated FM; this topic was the research hotspot of FM, although they were published in recent years. Second, two articles were expert consensus, which can attract the attention of researchers quickly. Third, two articles focused on the COVID-19-associated FM, which may be attributed to the COVID-19 pandemic. Finally, the 18th article was a review, which usually has high citations.

### Author analysis

To identify important contributors, the author’s information was obtained through author analysis. In addition, the main citation network could be summarized through the co-citation network analysis, and the research focus among authors could be analyzed. In addition, different name abbreviations of the same author were merged before analysis.

#### Influential authors

To a certain degree, the authors’ contribution could be evaluated by the following measurement indexes, including the TA published, TGCS, and GCSA. The top 20 contributors are shown in Table 4. A total of 4,411 authors were devoted to the research on FM, of which 54 authors had published more than five documents. Among all of the authors, Professor Klingel K published the most articles (12 articles), followed by Ammirati E (11 articles), Frigerio M (11 articles), and Izumi T (10 articles). In addition, the results showed that the number of articles by Moslehi JJ and Combes A was lower, but they were cited with high frequency. In addition, this indicated that they were very popular and with high attention.

**TABLE 4:** Top 20 contributing authors in terms of articles.

Rank	Author	Affiliation	TA	TA (%)	TGCS	GCSA
1	Klingel K	University Hospital Tubingen	12	1.7	327	27.3
2	Ammirati E	Ospedale Niguarda Ca’ Granda	11	1.5	374	34.0
3	Frigerio M	Ospedale Niguarda Ca’ Granda	11	1.5	359	32.6
4	Izumi T	Kitasato University	10	1.4	414	41.4
5	Fujita T	National Cerebral and Cardiovascular Center	9	1.2	43	4.8
6	Wang Y	Shandong University	9	1.2	264	29.3
7	Cipriani M	Ospedale Niguarda Ca’ Granda	8	1.1	298	37.3
8	Cooper LT	Mayo Clinic	8	1.1	299	37.4
9	Kobayashi J	National Cerebral and Cardiovascular Center	8	1.1	42	5.3
10	Minami K	National Cerebral and Cardiovascular Center	8	1.1	126	15.8
11	Yanase M	National Cerebral and Cardiovascular Center	8	1.1	41	5.1
12	Combes A	Hopital La Pitie Salpetriere	7	1.0	842	120.3
13	Fukushima N	National Cerebral and Cardiovascular Center	7	1.0	22	3.1
14	Ito M	Mie University	7	1.0	67	9.6
15	Kato S	University Ryukyus	7	1.0	118	16.9
16	Moslehi JJ	Vanderbilt University	7	1.0	1844	263.4
17	Seguchi O	National Cerebral and Cardiovascular Center	7	1.0	37	5.3
18	Wang DW	Huazhong University Science and Technology	7	1.0	39	5.6
19	Wang J	Shandong University	7	1.0	48	6.9
20	Asaumi Y	National Cerebral and Cardiovascular Center	6	0.8	160	26.7

TA, total articles; TGCS, total global citation score; GCSA, global citation score per article, which is the TGCS divided by the TA.

#### Author co-citation network analysis

**FIGURE 8:**
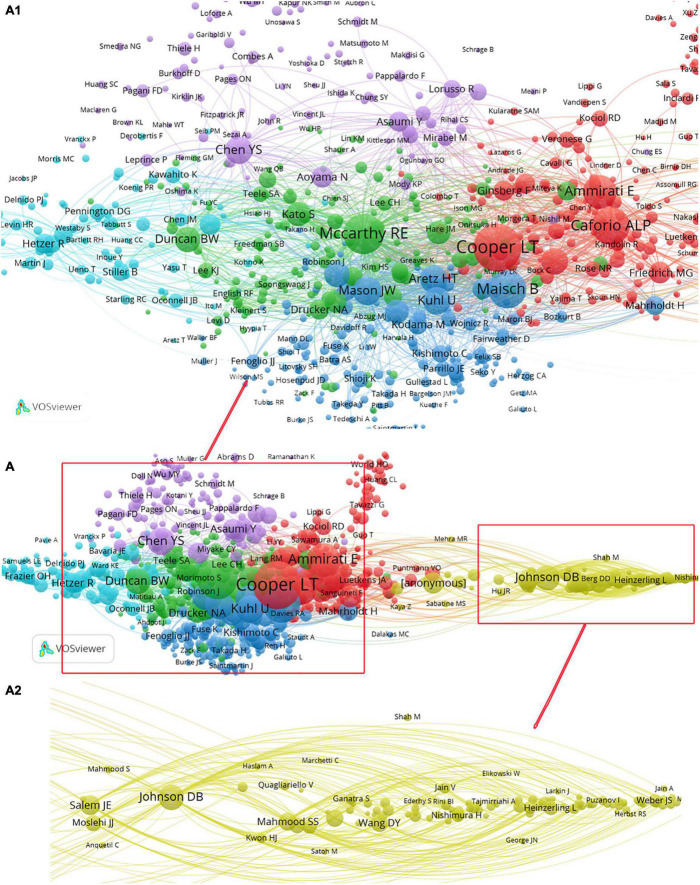
Author co-citation network analysis in the field of “fulminant myocarditis” research. **(A-1,A-2)** are partially enlarged views of **(A)**.

Among the 4,411 authors, 264 authors (6.0%) were cited not less than 100 times, 101 authors (2.3%) were cited at least 300 times, and 76 authors (1.7%) were cited not less than 500 times. To present the citation relations among authors, the ACA was carried out in this study, which was achieved by analyzing the references through BibExcel and VOSviewer software. After the ACA, authors’ names of the same color formed a cluster. The results of the authors’ co-citation network are presented in Figure 8, and there were six clusters. In addition, the core-authors at the center of the six clusters could be identified by the largest circle size or font size, and they were Cooper LT, Mccarthy RE, Maisch B, Chen YS, Acker MA, and Johnson B, which indicated that they made more contributions on this field.

## Discussion and managerial implications of the study

Fulminant myocarditis is a sudden-onset and life-threatening disease (3). Researchers have paid much attention to FM, including the diagnosis (11), therapy (53), and the relationship with other diseases (8). In the past three decades, the number of articles related to FM has been increasing. To better learn the research trends of FM, the bibliometric analysis was carried out, which could contribute to capturing the hot topic and research gap so that the researchers can gain the information quickly.

The first article about FM was published in 1985. Overall, the publications have been with fluctuating growth. In addition, the number of articles has grown rapidly since 2019, which might attribute to the international meeting related to FM held in 2019 (54). In the research about FM, 71 countries have concentrated, and they cooperate. The United States has played an important role in this area, publishing the most articles. In addition, 50% of the productive journals were from the United States. Japan played a second role in the number of articles, followed by China, Germany, and France.

The co-occurrence of keywords was analyzed to understand the research content better. The results indicate that there were four main topics in the area of FM: (I) Concentrate on the application of mechanical circulatory support to patients diagnosed with FM, especially for those with cardiogenic shock (55, 56). In addition, children with FM have been paid much attention to (57, 58). (II) Analyze diagnosis methods of FM, such as the endomyocardial biopsy (59), cardiac magnetic resonance (60), and the clinical manifestations (61). (III) Explore the etiology and pathogenesis of myocarditis, and the virus infection was the most popular mechanism, such as COVID-19 (62), parvovirus b19 (63), and influenza virus (64, 65). (IV) Monitor the ICI-associated with myocarditis (6, 66).

To further understand the topic, the research content of the top three prolific authors, the first three highly cited authors, and the core authors of co-citation network analysis were analyzed.

Klingel K, Ammirati E, Frigerio M, and Izumi T were identified as the top four prolific authors. Klingel K was the most productive author in this area, whose research area could be classified into two categories. On the one hand, the pathogenesis of FM has been paid much attention to since 2001. For example, Klingel K explored the mechanism of the parvovirus B19 (67), human herpesvirus 6 (67), and coxsackievirus B3 (68) causing FM. On the other hand, the therapy of FM has been kept an eye on since 2008, such as the role of Artesunate in the treatment of human herpesvirus 6B myocarditis (69), the protective role of effective chemokine secretion by dendritic cells and expansion of cross-presenting CD4(−)/CD8(+) in the coxsackievirus myocarditis (70), and mechanical circulatory support (71). Ammirati E and Frigerio M were the second productive authors, and their research hotspots were similar. They both paid attention to the prognosis and outcome of FM. For example, they were concerned about the outcomes of patients presenting with life-threatening ventricular arrhythmias (72) and explored the survival difference between FM and NFM (73). In addition, they both paid attention to some special cases, such as the OC43 subtype coronavirus-associated FM (7) and FM masked by acalculous cholecystitis (74). Compared with Ammirati E, Frigerio M also paid attention to mechanical circulatory support (75). Izumi T was the third productive author, who mainly kept an eye on the etiology, therapy, and outcome of FM, such as the mechanism of giant-cells associated myocarditis (76), the therapeutic effect of the cardiopulmonary support system for FM (77), and long-term prognosis of patients diagnosed with FM surviving from cardiopulmonary support (78). In addition, Izumi T also paid attention to the relationship between the influenza pandemic and FM (9). Puzanov I, Moslehi JJ, and Ernstoff MS were the top three highly cited authors. Their research area was extremely similar, and they all paid much attention to ICI-associated with myocarditis (6, 35). In addition, the publishing time of their article ranges from 2016 to 2021, which means that researchers have paid much attention to this area in the recent 5 years. Cooper LT, Mccarthy RE, Maisch B, Chen YS, Acker MA, and Johnson B were identified as the important contributors in this area by co-citation network analysis. Cooper LT mainly concentrated on the diagnosis, therapy, and outcome of giant cell myocarditis (59, 79). Mccarthy RE is concerned about the outcome of FM as compared with NFM (36). Maisch B kept an eye on etiology-directed immune therapies for myocarditis (80). The interest of Chen YS and Acker MA was similar; they both paid attention to the application of mechanical circulatory support in the treatment of FM (44, 81). Johnson B had an interest in the early recognition, clinical manifestations, and interventions of ICI-associated with FM (6, 37).

To a large extent, the research areas of key authors were similar to the results of keyword co-occurrence, the theme of the top 10 cited articles. With the understanding of FM, the research content has gradually been deepened. In addition, the recognition ability has been also improved. For example, the researchers have identified a new etiology of FM in recent years, that is, ICI (53). Although ICI is an important therapy for cancer patients, it can have serious side effects on patients, which can lead to FM (3). From the above analysis, we found that researchers have paid close attention to this area, which may have a significant influence on cancer patients. In addition, researchers have been continuing in exploring the diagnosis, therapy, and outcome of FM. In addition, they have paid attention to etiology-directed management (80). Researchers will have a better understanding of FM with the deepening of research.

This study has managerial implications for academia and researchers, and it has analyzed the research area of FM from the bibliometric perspective, which could help researchers have a holistic understanding of this area under the challenge of more articles being published. The research results help us learn the paper publishing trends, the countries and institutions participating in this area, the main journals, influential authors, the research trends, and hotspots of this area. According to the results, researchers can quickly identify the content they are interested in. For example, it can help authors choose a journal that is suitable for them when submitting their manuscript according to the journal analysis, and they also can find the potential cooperator according to the author’s analysis. In addition, researchers can quickly catch the research hotspots, which can guide their research. In general, this study can provide a reference for researchers to some extent.

This study provides a systematic analysis for us. But, notably, there are some limitations needed to be illustrated. First, the only information source for this study was from the WoS database, and the documents not covered by WoS were not involved. Second, the data were confined to articles published in English only. Finally, there may be a gap between the actual research conditions and the results of bibliometric analysis because of certain time factors. For example, the database updates the study continuously.

## Conclusion

Fulminant myocarditis can bring a serious threat to an individual’s life. Researchers are concerned about the disease, whether the etiology or the therapy. In addition, the diagnosis and recognizing ability have been also paid attention to. To have a comprehensive understanding of FM, this study carried out a bibliometric analysis of the study data. The results depicted the contributor authors, the hot topic, the participating countries, etc., which could provide a guideline for researchers. The results will deepen researchers’ understanding and lay the foundation for them to carry out research in the future.

This study showed that the following points should be paid high attention to in the future. First, productive countries should develop more international cooperation. Second, authors should make more contributions to FM in the future, because only 54 of 4,411 authors published 5 more documents. Third, researchers can pay more attention to ICI-associated myocarditis, research progress on etiology, diagnosis, therapy, and outcomes of FM, such as the new virus infection, the mechanical support option, and etiology-directed management.

## Data availability statement

The original contributions presented in this study are included in the article/supplementary material, further inquiries can be directed to the corresponding author.

## Author contributions

WMY, XFH, ZZW, and XYH wrote the main manuscript text. WMY, XYH, LJL, and JC prepared the Figures 1–8. WMY, XYH, and GZ collected, processed, and analyzed the data. All authors reviewed the manuscript.

## Footnotes

1
http://lert.co.nz/map/
